# Inactivation of Prions and Amyloid Seeds with Hypochlorous Acid

**DOI:** 10.1371/journal.ppat.1005914

**Published:** 2016-09-29

**Authors:** Andrew G. Hughson, Brent Race, Allison Kraus, Laura R. Sangaré, Lori Robins, Bradley R. Groveman, Eri Saijo, Katie Phillips, Luis Contreras, Virkamal Dhaliwal, Matteo Manca, Gianluigi Zanusso, Daniel Terry, Jeffrey F. Williams, Byron Caughey

**Affiliations:** 1 Laboratory of Persistent Viral Diseases, Rocky Mountain Laboratories, NIAID, NIH, Hamilton, Montana, United States of America; 2 Department of Epidemiology, University of Washington, Seattle, Washington, United States of America; 3 Department of Physical Sciences, University of Washington, Bothell, Washington, United States of America; 4 Department of Neurosciences, Biomedicine and Movement Sciences, Policlinico G.B. Rossi, University of Verona, Verona, Italy; 5 Briotech Inc., Woodinville, Washington, United States of America; University of Alberta, CANADA

## Abstract

Hypochlorous acid (HOCl) is produced naturally by neutrophils and other cells to kill conventional microbes *in vivo*. Synthetic preparations containing HOCl can also be effective as microbial disinfectants. Here we have tested whether HOCl can also inactivate prions and other self-propagating protein amyloid seeds. Prions are deadly pathogens that are notoriously difficult to inactivate, and standard microbial disinfection protocols are often inadequate. Recommended treatments for prion decontamination include strongly basic (pH ≥~12) sodium hypochlorite bleach, ≥1 N sodium hydroxide, and/or prolonged autoclaving. These treatments are damaging and/or unsuitable for many clinical, agricultural and environmental applications. We have tested the anti-prion activity of a weakly acidic aqueous formulation of HOCl (BrioHOCl) that poses no apparent hazard to either users or many surfaces. For example, BrioHOCl can be applied directly to skin and mucous membranes and has been aerosolized to treat entire rooms without apparent deleterious effects. Here, we demonstrate that immersion in BrioHOCl can inactivate not only a range of target microbes, including spores of *Bacillus subtilis*, but also prions in tissue suspensions and on stainless steel. Real-time quaking-induced conversion (RT-QuIC) assays showed that BrioHOCl treatments eliminated all detectable prion seeding activity of human Creutzfeldt-Jakob disease, bovine spongiform encephalopathy, cervine chronic wasting disease, sheep scrapie and hamster scrapie; these findings indicated reductions of ≥10^3^- to 10^6^-fold. Transgenic mouse bioassays showed that all detectable hamster-adapted scrapie infectivity in brain homogenates or on steel wires was eliminated, representing reductions of ≥~10^5.75^-fold and >10^4^-fold, respectively. Inactivation of RT-QuIC seeding activity correlated with free chlorine concentration and higher order aggregation or destruction of proteins generally, including prion protein. BrioHOCl treatments had similar effects on amyloids composed of human α-synuclein and a fragment of human tau. These results indicate that HOCl can block the self-propagating activity of prions and other amyloids.

## Author Summary

Many serious diseases have been linked to pathogenic states of various proteins. These naturally occurring proteins can be corrupted to form aggregates such as prions and amyloids that propagate in and between tissues by acting as seeds that convert the normal form of the protein into more of the pathological form. For example, corrupted prion protein can cause fatal transmissible neurodegenerative diseases such as Creutzfeldt-Jakob disease in humans, chronic wasting disease in cervids and bovine spongiform encephalopathy. Other amyloid-forming protein aggregates are pathogenic in Parkinson’s, Alzheimer’s, and other diseases. The fact that prions and amyloids are composed predominantly of tough, tightly packed proteins makes them unusually resistant to conventional microbial disinfection procedures. Infectious prions can persist indefinitely in, or on, a variety of materials such as tissues, fluids, tools, instruments, and environmental surfaces, making it important to identify decontaminants that are effective without being dangerous or damaging. Here we show that hypochlorous acid, a disinfectant that is produced naturally by certain cells within the body, has strong anti-prion and anti-amyloid activity. We find that a non-irritating and broadly applicable hypochlorous acid preparation can disinfect prions in tissue homogenates and on stainless steel wires serving as surrogates for surgical instruments.

## Introduction

Prion diseases, or transmissible spongiform encephalopathies (TSEs), are fatal and untreatable neurodegenerative diseases. In humans, prion diseases include sporadic, variant and genetic forms of Creutzfeldt-Jakob disease (sCJD, vCJD and gCJD) as well as a number of other disorders [1–3]. Prion diseases of other species include classical bovine spongiform encephalopathy (C-BSE) [4], scrapie in sheep, goats [5] and rodents, and chronic wasting disease (CWD) of cervids [6, 7]. All mammalian prion diseases share an underlying molecular pathology that involves the conversion of the hosts’ normal form of prion protein, PrP^C^, to a misfolded, aggregated, infectious and pathological form, PrP^Sc^ [8, 9].

Compared to other types of pathogens, prions are unusual in that they lack a pathogen-specific nucleic acid genome, and tend to be particularly resistant to enzymatic, chemical, physical (eg. heat) or radiological inactivation [8, 10]. As a result, prions can resist complete inactivation under conditions that are typically used in medicine, the food industry, and agriculture to inactivate other types of pathogens. Current prion decontamination recommendations include incineration or harsh chemical treatments such as 1–2 N sodium hydroxide, 20–40% household bleach (20,000 ppm sodium hypochlorite) alone or, preferably, in combination with prolonged autoclaving to treat relevant materials or surfaces [10, 11]. Other effective treatments include enzymatic treatments with SDS [12], vaporized hydrogen peroxide [13] or 4% SDS in 1% acetic acid at 65–134°C [14, 15]. Environ LpH^™^ also inactivates prions [16, 17] but the active formulation of this acidic phenolic disinfectant has been removed from the market. Most, if not all, of the above treatments are potentially hazardous to the user and/or incompatible with various purposes. Thus, more safely and broadly applicable anti-prion reagents are needed.

In humans, iatrogenic transmission of prion disease has occurred through the use of contaminated instruments, transplanted tissues or tissue extracts [2, 18]. The risk alone of iatrogenic transmission has been highly problematic when surgical procedures have been performed on patients who were later discovered to have sCJD [18]. Because routine disinfection procedures are not likely to be fully adequate for prions, the reuse of potentially contaminated tools or instruments on subsequent patients presents transmission risks. Prion decontamination is also a significant concern in autopsies and mortuary functions. In livestock, prion diseases can be spread via contaminated feeds and/or the environment [19, 20]. In cervids, the rampant spread of CWD threatens both captive and free-ranging populations in North America, Asia, and now Europe. With BSE, at least, there is also apparent zoonotic risk associated with contaminated beef and its handling in slaughter houses. Thus, prion disinfectants are needed that can be used routinely on potentially contaminated tools, instruments, and environmental surfaces to reduce the risks of prion transmission.

Here we have tested a synthetic preparation of hypochlorous acid (HOCl), a reactive oxygen species that is produced naturally *in vivo* to inactivate pathogens. Synthetic formulations containing HOCl have been shown to kill bacteria, viruses, fungi and protozoans [21–23]. HOCl is the conjugate acid of hypochlorite, the sodium salt of which is the main component of household hypochlorite bleach. In concentrations recommended for prion inactivation, hypochlorite bleach is corrosive and highly basic, i.e. pH ≥~12, whereas HOCl solutions are weakly acidic, i.e. pH 3.7–6.3, and apparently safe for contact with skin and mucous membranes. For example, at least some HOCl formulations are used in cosmetics and topical skin treatments for humans and domestic animals (e.g. www.briotechinternational.com) and/or have strong antimicrobial activity at non-cytotoxic concentrations (e.g. [24]). Furthermore, electrolytically generated HOCl is acknowledged to be both powerful and benign enough to meet USDA standards for sanitation and safe food contact without need for rinsing (FSIS Directive 7120.1, Rev. 36, 6/29/16. US Dept. of Agriculture, pp 31–32). Many studies have described anti-microbial activities of HOCl, but only one has raised the possibility of anti-prion activity. In that study, a cycle of sonications and/or washes with electrolyzed basic (pH 11.9) and acidic (pH 2.7) water, with the latter presumed to contain HCl and HOCl, inactivated prions by ≥1 log_10_ on steel wires [25, 26]. However, the role of HOCl in the anti-prion activity of this cyclic treatment remains unclear because, firstly, Cl_2_ is also a prominent oxidizing species present in aqueous free-chlorine solutions at pH 2.7 [27]; and secondly, the pH 11.9 step may have been important given that basic solutions can have anti-prion activity [13, 16, 28, 29].

For the present study, we evaluated the anti-prion effects of a single unsonicated treatment with a mildly acidic, electrochemically-activated HOCl formulation (BrioHOCl) using both mouse bioassays [30, 31] and real time quaking-induced conversion (RT-QuIC) assays [32–36]. Animal bioassays are the gold standard tests for prion infectivity but are also costly, animal-intensive, and time-consuming—typically requiring months-years. RT-QuIC assays exploit the inherent self-propagating activity of prions by measuring a sample’s ability to seed the *in vitro* conversion of recombinant PrP^C^ (rPrP^C^) into amyloid fibrils that enhance the fluorescence of thioflavin T (ThT) [32, 37]. Detection of RT-QuIC seeding activity correlates strongly with the presence of prion infections in mammalian hosts [32–49]. These assays are not only at least as sensitive as bioassays, but are also much more rapid, high throughput and cost-effective. Thus, our strategy was to first test effects of HOCl and other conventional anti-prion reagents using RT-QuIC, and then confirm any observed effects on infectivity using bioassays. Because of concerns about iatrogenic transmission of prion diseases via contaminated surgical instruments [18], and the tenacious binding and infectivity of prions bound to stainless steel [50, 51], we have not only tested HOCl inactivation of prions in brain homogenates (BH), but also prions on stainless steel wire as a surrogate for surgical instruments. The latter strategy has been employed previously for the evaluation of other disinfectants [12–15, 25, 26, 52]. To investigate whether HOCl might also inactivate other types of self-propagating amyloid seeds, we have also tested effects of BrioHOCl on amyloid seeds composed of human α-synuclein (α-syn) and tau. Aggregated forms of α-syn and tau are prominent pathological features of various proteinopathies including Parkinson’s and Alzheimer’s diseases respectively.

## Results

### Characterization of the HOCl preparation used for anti-prion testing

BrioHOCl is produced by a proprietary process involving electrochemical activation of saline solutions. Although some electrochemically activated saline preparations may contain various amounts of HOCl, OCl^-^ (hypochlorite), HCl and Cl_2_, analysis of BrioHOCl preparations by Raman spectroscopy has only indicated the presence of HOCl (S1 Fig). Application of BrioHOCl preparations with various levels of free (active, available) chlorine showed strong microbicidal activity against multiple bacterial and fungal pathogens, including spores of *Bacillus subtilis* and *Aspergillus* (S1 Table).

### Inactivation of hamster scrapie prion seeding activity in brain homogenates by HOCl

**Fig 1:**
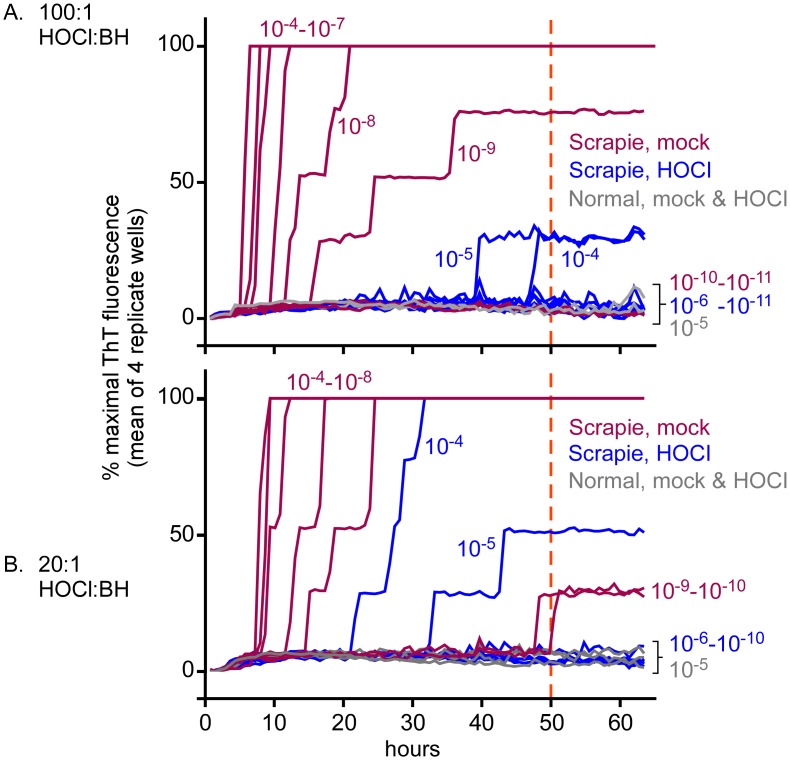
Inactivation of prion seeding activity in hamster ScBH by BrioHOCl. ScBH was pretreated for 1 h in BrioHOCl at 100:1 v/v BrioHOCl to 10% BH (panel A, blue), 20:1 BrioHOCl (panel B, blue) or corresponding saline treatments (panels A and B, red). Similar BrioHOCl and saline pretreatments of NBH are indicated in gray. Resulting samples were then subjected to serial 10-fold dilutions and RT-QuIC analysis was performed with hamster (90–231) rPrP^C^ substrate using 2 μl per well of the indicated tissue dilutions as reaction seeds. The dilutions noted refer to the final dilution of original brain mass used to seed the reaction. The orange dashed line indicates the 50-h cutoff time at which seeding activity was quantified by end-point dilution under these assay conditions, after which unseeded control reactions occasionally gave false-positive reactions (see Material and Methods). Each trace represents the average ThT fluorescence of 4 technical replicate wells normalized between baseline and maximal signal and graphed as a function of time.

For an initial indication of potential anti-prion activity, we tested effects of BrioHOCl on the RT-QuIC seeding activity in scrapie brain homogenate (ScBH) from clinically ill hamsters. We first tested the tolerance of the RT-QuIC assay for a BrioHOCl preparation with ~300 ppm Cl, 0.98% NaCl, pH 4.25, and 1138 mV oxidation-reduction potential. We saw no interference with positive control RT-QuIC reactions seeded with ScBH when the final concentration of the BrioHOCl added directly to the RT-QuIC reaction was ≤0.1% (S2 Fig). To test the effects of preincubation of the ScBH with HOCl, we mixed BrioHOCl 100:1 (v/v) with 10% ScBH, incubated for 1 h at room temperature, and used sufficiently diluted, treated ScBH mixtures to seed RT-QuIC reactions. Fig 1 shows the average ThT fluorescence readings from 4 replicate RT-QuIC reaction wells. Mock (saline)-treated ScBH samples gave strong positive responses in all, or 3 of 4, replicate RT-QuIC reactions when seeded with ScBH dilutions of 10^−4^ to 10^−9^ (see Materials and Methods), indicating a dynamic range for the assay of ≥10^5^ (Fig 1A, red traces). In contrast, with the BrioHOCl-treated ScBH samples (Fig 1, blue traces), no positive responses were seen with the same range of tissue dilutions prior to our 50-h cutoff (orange dashed line) except for single positive reactions (out of 4) at ~40 h from the 10^−4^ and 10^−5^ tissue dilutions. According to our standard criteria, a single positive well out of 4 replicates is not regarded as positive. Thus, these results indicated that the 100:1 BrioHOCl treatment reduced the scrapie seeding activity by ≥~100,000 fold. No positive responses were obtained in negative control reactions seeded with normal (uninfected) brain homogenate (NBH). Treatments with 20:1 BrioHOCl:10% ScBH reduced the scrapie seeding activity by ~10,000-fold (Fig 1B, blue traces). Thus, the 100:1 treatment was more effective than the 20:1 treatment. Similar results were obtained in a second independent experiment performed with a different batch of BrioHOCl.

### Inactivation of sCJD, vCJD, C-BSE, CWD and sheep scrapie seeding activity by BrioHOCl

**Fig 2:**
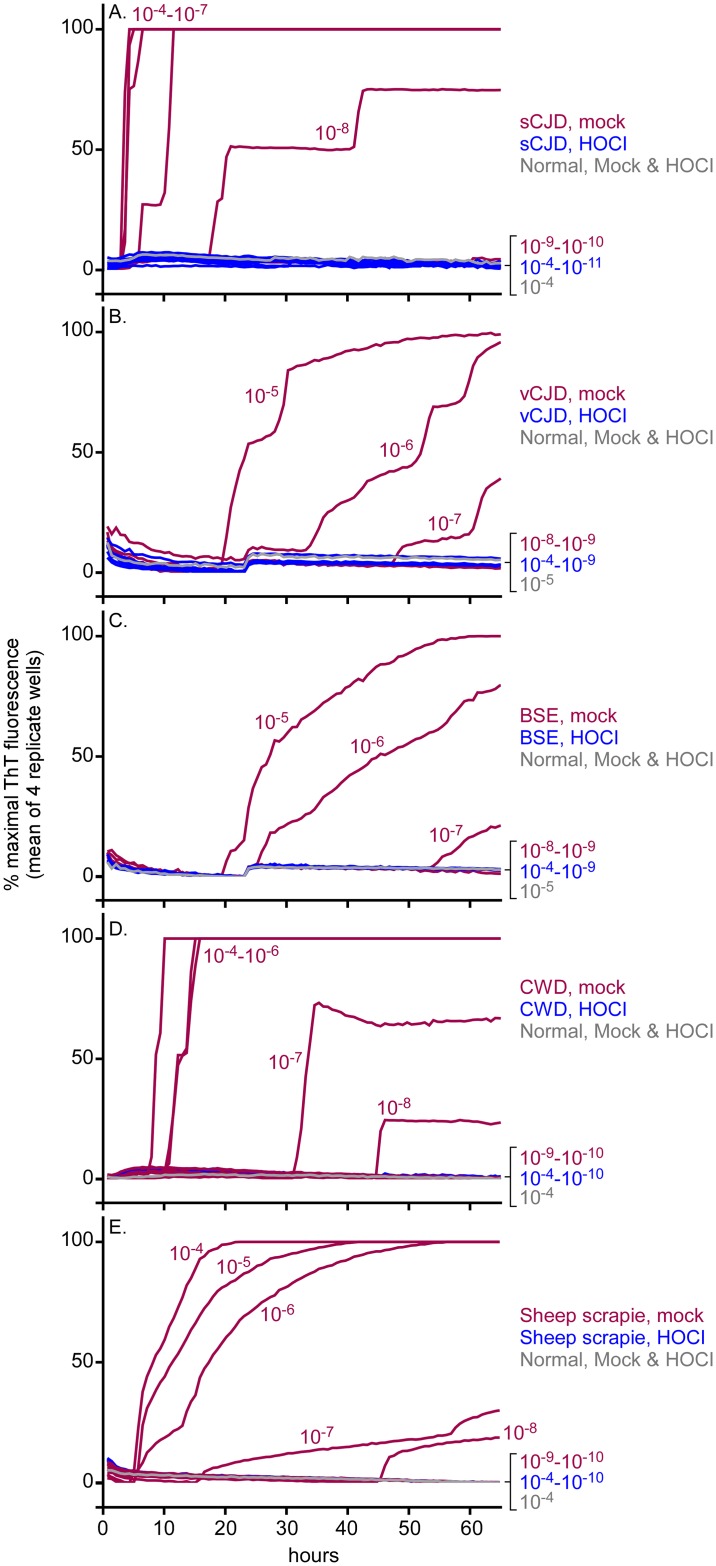
Inactivation of sCJD (A), vCJD (B), BSE (C), CWD (D) & sheep scrapie (E) seeding activity in brain homogenates by BrioHOCl. The indicated BH samples (10%) were pretreated for 1 h with 100 volumes of BrioHOCl (blue), or saline (red) as a mock treatment control. Similar HOCl and saline pretreatments of NBH are indicated in gray. Resulting samples were then subjected to serial 10-fold dilutions and RT-QuIC analysis was performed using 10^−4^ through 10^−11^ tissue dilutions as indicated. Hamster (90–231) rPrP^C^ was used as substrate for the sCJD and CWD reactions while chimeric hamster-sheep rPrP^C^ was used as substrate for the vCJD, BSE and sheep scrapie reactions. Each trace represents the average normalized ThT fluorescence of 4 replicate wells.

We performed similar experiments to test the effects of BrioHOCl on prion seeding activity associated with sCJD, vCJD, C-BSE, CWD and classical sheep scrapie. Because the starting titers of seeding activity of these prions in brain homogenates were lower than those of hamster ScBH, the dynamic range of these RT-QuIC assays was lower. However, no seeding activity was detected in these types of brain homogenates with 100:1 BrioHOCl:10% BH treatments for 1h (Fig 2A–2E). These results indicated that this BrioHOCl treatment reduced the seeding activity of each of these types of prion by at least 1,000–10,000 fold.

### Effects of bleach, NaOH and Environ LpH^™^ on hamster scrapie seeding activity

**Fig 3:**
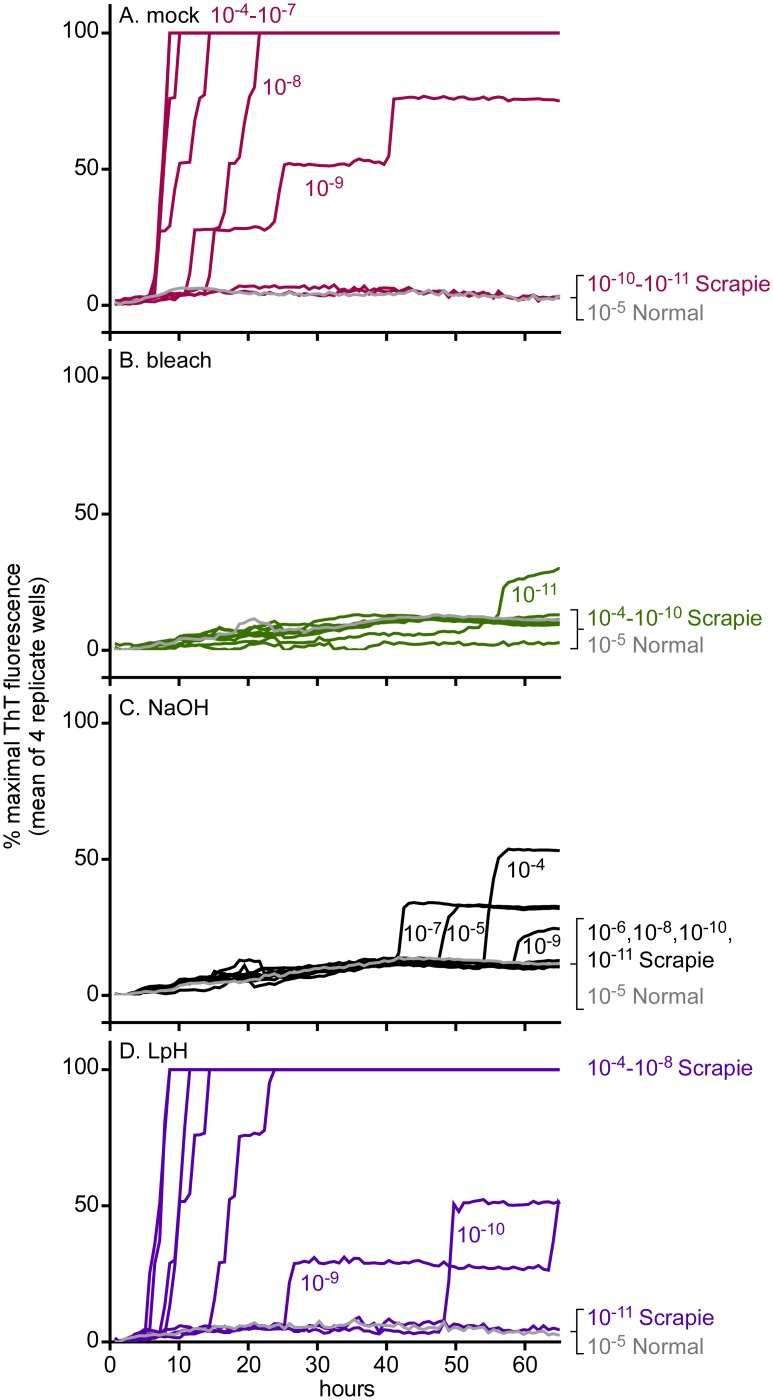
Effects of bleach, NaOH and Environ LpH^™^ on hamster scrapie seeding activity. ScBH was pretreated for 1 h in (A) saline (mock disinfectant) (red), (B) 40% bleach (2.4% hypochlorite) (green), (C) 1 N NaOH (black) or (D) 2% Environ LpH ^™^ (purple) at a ratio (v/v) of 100:1 disinfectant to 10% ScBH. Similar disinfectant pretreatments of NBH are indicated in gray. Resulting samples were then subjected to serial 10-fold dilutions and RT-QuIC analysis was performed with hamster (90–231) rPrP^C^ substrate using the designated tissue dilutions as seeds. Each trace represents the average normalized ThT fluorescence of 4 replicate wells.

To compare the effects of BrioHOCl with established anti-prion reagents, we performed similar end-point dilution RT-QuIC titrations on hamster ScBH samples tested with 40% bleach (2.4% hypochlorite), 1 N NaOH, or 2% Environ LpH^™^ for 1 h. Similar to the above observations with BrioHOCl (Fig 1A) both the bleach and NaOH showed >100,000-fold reductions in hamster scrapie seeding activity (Fig 3B and 3C). Surprisingly, given its reported effects on prion infectivity [13, 16] and our own bioassay data below, the Environ LpH^™^ had no effect on prion seeding activity as measured in the RT-QuIC (Fig 3D).

### Bioassay of scrapie prion infectivity in HOCl-treated brain homogenates

Having seen that BrioHOCl solutions can strongly inactivate multiple types of prion seeding activity as measured by RT-QuIC assays, we then tested effects on hamster scrapie infectivity directly by performing bioassays in transgenic mice (tg7) that overexpress hamster PrP [31]. For comparison, we also bioassayed effects of bleach, NaOH and Environ LpH^™^ using the same ScBH samples that were treated and assayed by RT-QuIC as shown in Fig 3. Serial 10-fold dilutions of the disinfectant- or saline-treated (100:1, 1h) ScBH samples were inoculated into groups of 4 tg7 mice to establish infectivity titers. As expected, the saline-treated sample retained high levels of infectivity with a calculated titer of 10^6.75^ 50% lethal doses (LD_50_) per mg of brain (Table 1) similar to previous 263K scrapie titers in hamster brain (e.g. [32]). In contrast, no mice developed scrapie in any of the groups that were inoculated with ScBH treated with BrioHOCl, 40% bleach, 1 N NaOH or 2% Environ LpH^™^ (100:1 v/v for 1 h; Table 1). To estimate the maximum infectivity titer that could have remained in the treated samples without causing disease in any of the inoculated mice, we assumed that a 10-fold more concentrated sample would have caused disease in 100% of the mice. This “worst-case” assumption should be conservative, meaning that the actual titers in the disinfectant-treated samples are actually lower than reflected by the numbers indicated. This analysis showed that BrioHOCl reduced the titer by at least 10^5.75^-fold compared to the saline-treated controls (Table 1). Because the samples treated with the other disinfectants had to be diluted 100-fold more than the HOCl-treated samples prior to inoculation to avoid acute toxic effects, we were only able to show titer reductions of 10^3.75^ fold relative to the saline-treated control for NaOH, bleach, and Environ LpH^™^.

**Table 1:** Mouse bioassay of hamster scrapie brain homogenates after treatment with disinfectants.

Treatment	Dilution of scrapie brain homogenate after treatment^a^								
	10^−3^	10^−4^	10^−5^	10^−6^	10^−7^	10^−8^	10^−9^	10^−10^	Titer^b^
Saline	nt	4/4^c^ [56]	4/4 [66]	4/4 [66.8]	4/4 [77.3]	2/4 [107.5]	1/4 [101]	0/4	6.75
HOCl	0/4^d^	0/4	0/4	0/4	0/4 ^d^	0/4	nt	nt	≤ 1
NaOH	nt	nt	0/4	0/4	0/4	0/4	nt	nt	≤ 3
Bleach	nt	nt	0/4 ^d^	0/4	0/4 ^d^	0/4	nt	nt	≤ 3
LpH	nt	nt	0/4 ^d^	0/4	0/4	0/4	0/4 ^d^	nt	≤ 3

^a^ Scrapie brain homogenates (10^−3^ dilution) were exposed to different disinfectants or saline for 1 h at a 1:100 (v:v) ratio. Solutions were then further diluted for bioassay in tg7 mice. Each recipient mouse received 30μl of inoculum.

^b^ The calculated titer reported is the logLD_50_/mg of tissue

^c^ The numerator is the number of scrapie-positive mice, and the denominator is the number of mice inoculated. For groups with positive mice the average scrapie incubation period is provided. Mice that did not develop disease were observed for 200 days post inoculation (dpi).

nt = Not tested (see methods for explanation)

^d^ Indicates a mouse/mice in this group was/were euthanized between 73–186 dpi due to dermatitis, intercurrent disease or were found deceased. Brains from these mice were screened for TSE infection by RT-QuIC for prion seeding activity and by Western blot for protease-resistant PrP. No mice in these groups were diagnosed with scrapie.

### Inactivation of steel-bound prion seeding activity by HOCl, NaOH and bleach

**Fig 4:**
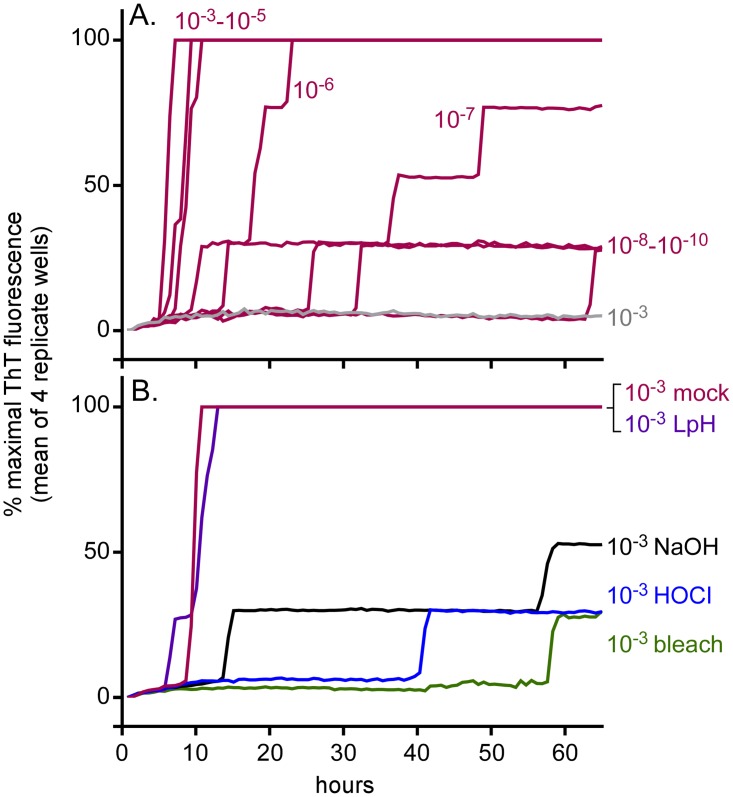
Inactivation of steel-bound prion seeding activity. A. RT-QuIC reaction wells were seeded with a 3–4 mm segment of stainless steel wire pre-coated with hamster scrapie (red) or normal BH (gray) at tissue dilutions of 10^−3^–10^−10^ as indicated. B. Wire segments pre-coated with ScBH at a 10^−3^ tissue dilution were submersed for 1 h in saline (mock disinfectant; red), BrioHOCl (blue), 40% bleach (2.4% hypochlorite; green), 1 N NaOH (black) or 2% Environ LpH (purple) as indicated prior to RT-QuIC analysis using hamster (90–231) rPrP^C^ substrate. Each trace represents the average normalized ThT fluorescence of 4 replicate wells.

We then addressed the possibility that BrioHOCl might inactivate prions that have been dried onto solid surfaces such as stainless steel. We initially tested whether steel-bound prions can be detected by RT-QuIC. Short 3–4 mm segments of stainless steel wire (n = 4 per dilution) were immersed in 10^−3^–10^−10^ dilutions of ScBH for 1 h, washed with PBS, dried, and then placed individually into RT-QuIC reaction wells. Consistent with a recent report [26], wires coated with as little as 10^−7^ dilutions of ScBH gave positive reactions in at least 3 of 4 replicate RT-QuIC reactions, whereas those coated with NBH gave no positive reactions (Fig 4A).

To test the effects of HOCl and other disinfectants on steel-bound prion seeding activity, wires coated with a 10^−3^ dilution of ScBH were immersed in BrioHOCl, 40% bleach, 1 N NaOH, 2% Environ LpH^™^, or saline for 1 h, rinsed, and added to RT-QuIC reactions. Although Environ LpH^™^ was again no more effective than PBS, the HOCl, NaOH and bleach treatments reduced the RT-QuIC responses to less than those seen from the mock (PBS)-treated wires coated with a 10^−7^ dilution of ScBH and similar to wires coated with 10^−8^–10^−10^ dilutions (compare Fig 4A and 4B). The latter dilutions were close to, or possibly beyond, the detection limit of the assay. These results provided evidence that the BrioHOCl, NaOH, and bleach treatments were similarly able to reduce the steel-bound scrapie seeding activity by at least 10,000-fold.

**Fig 5:**
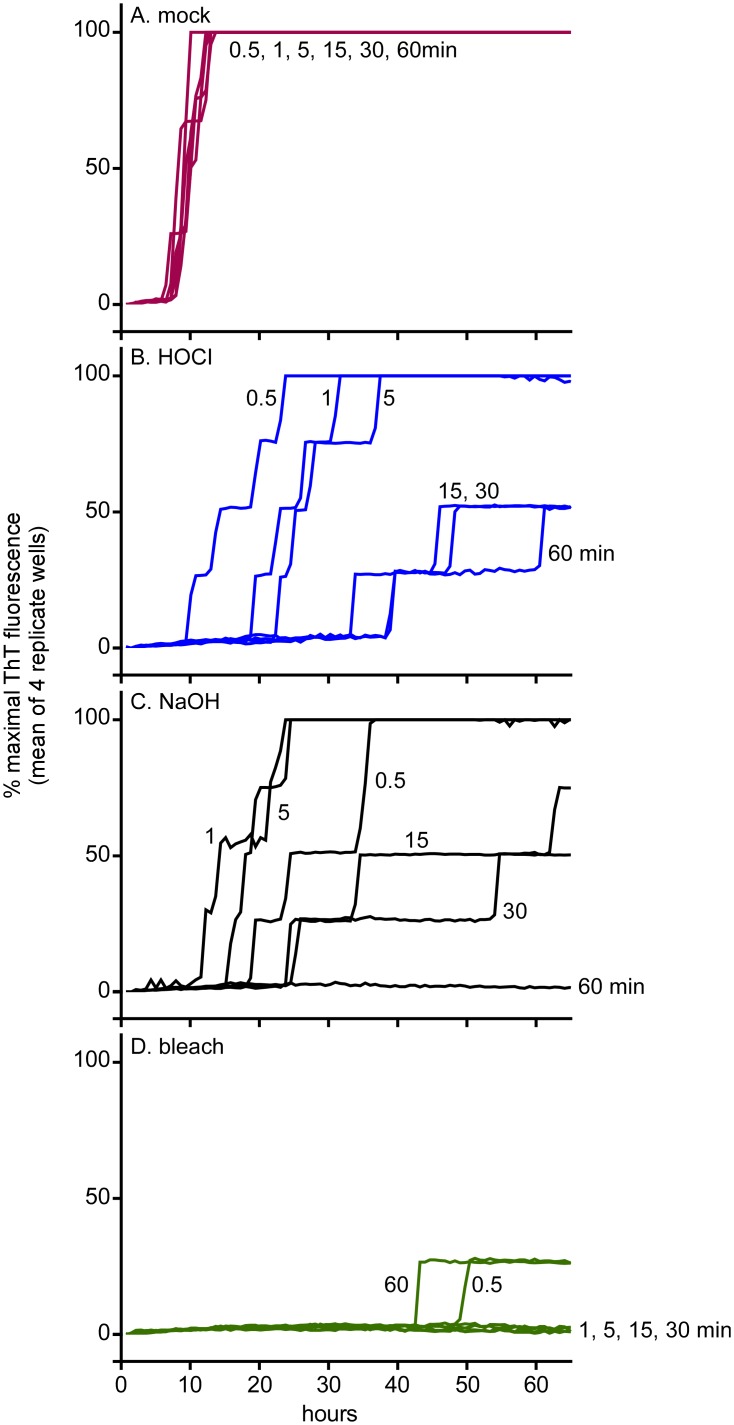
Effect of disinfectant exposure time on inactivation of hamster scrapie seeding activity on wires coated with ScBH. Stainless steel wire segments (3–4mm) pre-coated with hamster ScBH at a 10^−3^ tissue dilution were submersed in (A) saline (mock disinfectant), (B) BrioHOCl, (C) 1 N NaOH or (D) 40% bleach (2.4% hypochlorite) for 0.5–60 min prior to a quick rinse and RT-QuIC analysis using hamster (90–231) rPrP^C^ substrate. Each trace represents the average normalized ThT fluorescence of 4 replicate wells.

To determine the speed of prion seed inactivation by the BrioHOCl, bleach and NaOH, wires (n = 4 per treatment) were coated with a 10^−3^ dilution ScBH, rinsed and dried. The wires were then treated for 0.5–60 min and then rinsed briefly prior to being added to RT-QuIC reaction wells (Fig 5A–5D). The time-dependence of the effects of HOCl and NaOH were similar, with slower RT-QuIC responses on average from the 0.5 min treatment (Fig 5B and 5C), and further inactivation with more prolonged treatments. With both BrioHOCl, and NaOH, treatments of 30–60 min were required to maximize inactivation of seeding activities that were near the detection limit of the assay. In contrast, the bleach treatment gave near maximal inactivation within 0.5–1 min (Fig 5D).

### Bioassay of inactivation of steel-bound scrapie infectivity

To determine the effects of HOCl on steel-bound scrapie infectivity, we implanted single BrioHOCl-treated or mock-treated stainless steel wires into the brains of tg7 mice. Concurrently, we also tested bleach-, NaOH- and Environ LpH^™^-treated wires for scrapie infectivity. To measure the infectivity that could be bound and transmitted by wire fomites we implanted wires exposed to serial 10-fold dilutions of hamster ScBH (Table 2). All of the mice with wires exposed to 10^−3^ to 10^−6^ dilutions developed clinical scrapie. Unlike the results from the solution experiments, the disease incubation period did not consistently decrease with wires exposed to ScBH more concentrated than 10^−5^. This result suggested that the wires were limited in how much prion infectivity could be tightly bound in a manner that resists short washes in PBS. Importantly, none of the mice that received scrapie-coated wires treated with BrioHOCl, bleach or LpH, and only one treated with NaOH, developed prion disease.

**Table 2:** Bioassay of scrapie-coated wires after treatment with disinfectants.

Treatment	Dilution of 263K ScBH							
	10^−3^	10^−4^	10^−5^	10^−6^	10^−7^	10^−8^	10^−9^	10^−10^
None	4/4 ^b^ [99±18]	4/4 [90±10]	4/4 [90±4]	4/4 [144±7]	0/3	0/4	0/4	0/4
Saline	6/7 [96±14]	nt	nt	nt	nt	nt	nt	nt
HOCl	0/7	nt	nt	nt	nt	nt	nt	nt
NaOH	1/8 [137]	nt	nt	nt	nt	nt	nt	nt
Bleach	0/8	nt	nt	nt	nt	nt	nt	nt
LpH	0/8	nt	nt	nt	nt	nt	nt	nt

^a^ Steel wires were exposed to hamster 263K ScBH, then washed, dried and either untreated (None) or treated by immersion in saline or the designated disinfectants for 1 h. Following treatment wires were removed and allowed to dry. Each mouse was implanted intracerebrally with a single 3–4 mm wire.

^b^ The numerator is the number of scrapie-positive mice, and the denominator is the number of mice implanted. For groups with positive mice the average scrapie incubation period +/- SD is provided in brackets. Mice that did not develop disease were observed for 200 d.

### Comparison of BrioHOCl preparations with different free chlorine concentrations

**Fig 6:**
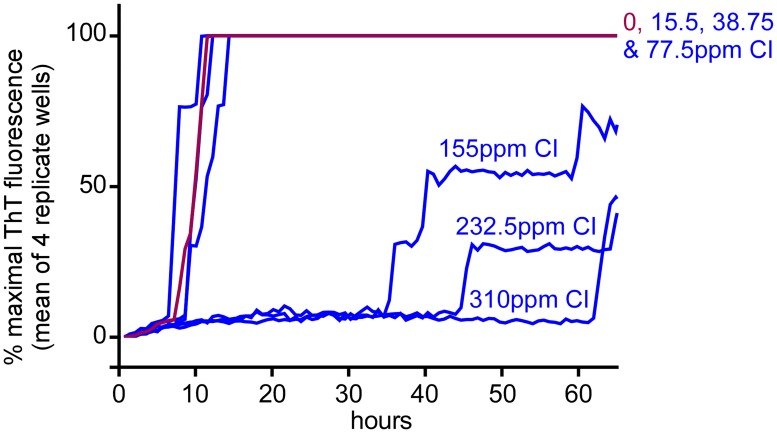
Effect of BrioHOCl free Cl concentration on scrapie seeding activity. Hamster ScBH was pretreated for 5 min in saline (mock disinfectant) (red) or BrioHOCl formulations (blue) containing the designated ppm of free Cl at a ratio (v/v) of 100:1 disinfectant to 10% ScBH. Resulting samples were then subjected to serial 10-fold dilutions and RT-QuIC analysis was performed with hamster (90–231) rPrP^C^ substrate using 10^−4^ through 10^−7^ tissue dilutions as seeds. For simplicity, only the 10^−6^ dilutions are shown here. See Table 3 for summary of all results. Each trace represents the average normalized ThT fluorescence of 4 replicate wells.

To test the effect of free (active) chlorine concentration on scrapie inactivation, we treated 10% ScBH with 100 volumes of dilutions of BrioHOCl (in 2% saline) with increasing Cl (15–310 ppm), and then compared the remaining scrapie seeding activity levels by RT-QuIC. We deliberately chose milder (5 min) treatment conditions that would only partially inactivate the prion seeding activity so that changes in inactivation efficiency could be differentiated more readily by end-point dilution RT-QuIC. Fig 6 shows the primary RT-QuIC data from 10^−6^ dilutions of treated ScBHs. Strong inhibition of seeding activity within this short exposure time was seen only with Cl concentrations of ≥160 ppm. Full end-point dilution analyses [32] of the treated ScBH preparations from 2 independent experiments confirmed that ≥155 ppm Cl was needed for >50 to 100-fold reductions of the concentrations of seeding doses resulting in 50% ThT-positive replicate reactions (SD_50_s) (Table 3). In the second series shown here dose-responses were determined using a sample set in which the pH was adjusted to the same starting level (3.9) with 100 mM HCl, so as to obviate any effects on the outcome attributable to the rise in pH on dilution (up to pH ~6.3) seen with the first series.

**Table 3:** RT-QuIC titration of ScBH treated with BrioHOCl with increasing available Cl.

Experiment 1			Experiment 2		
Active Cl	Log_10_SD_50_/mg brain	pH	Active Cl	Log_10_SD_50_/mg brain	pH
saline	>7.70	7.4	saline	>7.70	7.4
15.5 ppm Cl	>7.20	6.3	16 ppm Cl	>7.20	3.9
38.8 ppm Cl	>7.20	5.9	40 ppm Cl	>7.20	3.9
77.5 ppm Cl	>7.20	4.3	80 ppm Cl	>6.95	3.9
155 ppm Cl	5.45	3.9	160 ppm Cl	5.20	3.9
232 ppm Cl	4.20	3.9	240 ppm Cl	4.70	3.9
310 ppm Cl	<3.45	3.8	320 ppm Cl	<4.20	3.9

^a^ log_10_SD_50_/mg brain calculated from end-point dilution RT-QuIC experiments conducted at 42°C with the reaction well data collected at a 50 hour time point.

### Storage stability of BrioHOCl

Electrochemically activated HOCl preparations are metastable and may change over time during the “relaxation period” after production. Archived production samples from lots that contained ~300 ppm Cl at the time of manufacture declined to as low as 58 ppm over almost 3 years of warehouse storage at uncontrolled temperatures. Nonetheless, one of the oldest samples, with 80 ppm Cl at 34 months, still showed high efficacy vs. *Bacillus* spores [3.9 log removal value (LRV) in 15 seconds; S1 Table]. Oxidation-reduction potentials remained high throughout, some remaining unchanged over more than two years of storage; few declined more than 10%. Samples stored unsealed showed precipitous declines in Cl ppm, losing approximately 90% of their Cl content in six months.

**Fig 7:**
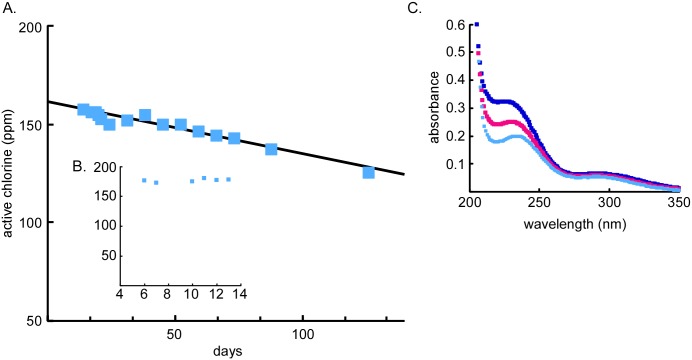
Chemical stability of BrioHOCl. A. Concentration of active chlorine as measured by iodometric titration (days 14–151). The data were fit to an exponential decay (y = 159.78e^(-0.00157x)^). B. Active chlorine concentrations over initial 13 days. C. UV-VIS measurements of samples after 10 (dark blue), 14 (pink), and 88 (light blue) days.

Sealed aliquots from a lot prepared specifically for study of stability remained unopened until a sample from each was titrated for active Cl. The results over the first 3 months are shown in Fig 7. From a starting concentration of 185 ppm the Cl showed no discernible pattern of change for about two weeks (Fig 7A and 7B), then settled into a slow and steady decline, allowing for computation of a half-life of 440 days. The HOCl UV absorbance peak at 238 nm in these samples showed a downward trend corresponding to the titratable Cl ppm decline (Fig 7C).

### Effect of BrioHOCl on PrP^Sc^ and other proteins

**Fig 8:**
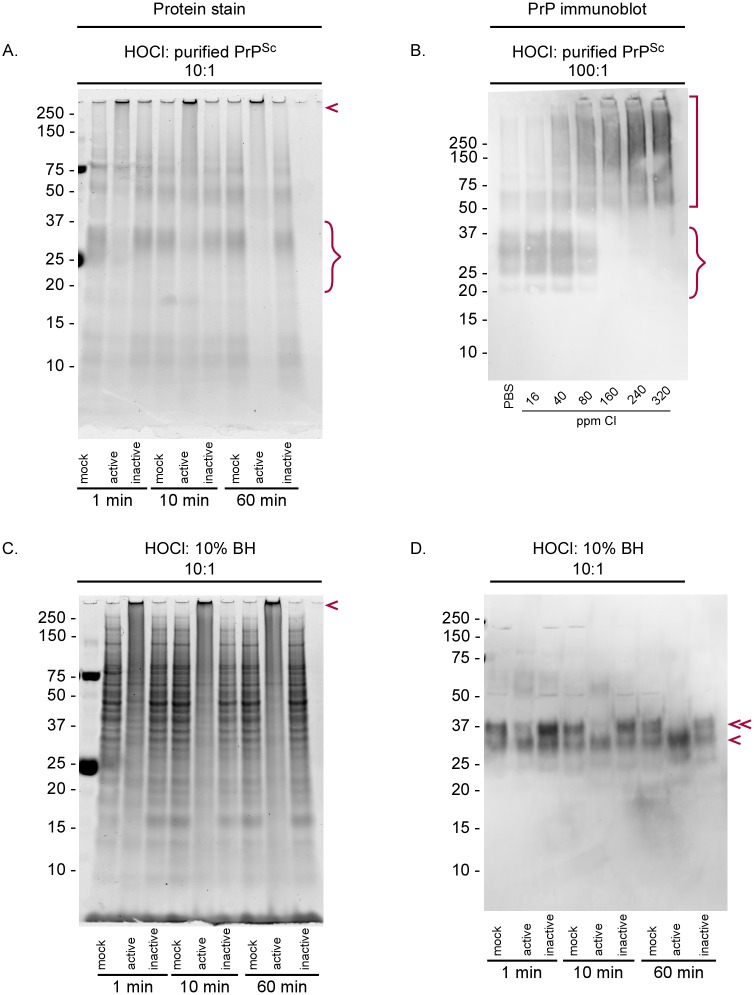
SDS-PAGE analysis of BrioHOCl treatments on purified PrP^Sc^ and ScBH. (A) Purified PrP^Sc^ was treated with 10 volume equivalents of saline (mock), active (260 ppm Cl) or inactive (30 ppm Cl) BrioHOCl solutions for 1, 10 or 60 min as indicated. BrioHOCl activity or inactivity was determined by its ability or inability to reduce prion-seeding activity in the RT-QuIC in other experiments. Samples were run on denaturing protein gels and visualized using Deep Purple total protein stain. PrP monomer (curly bracket) and multimeric aggregates (arrowhead) are marked. (B) Purified PrP^Sc^ (at 3 mg/mL) was treated for 5 minutes in saline (mock disinfectant) or BrioHOCl formulations containing the designated ppm free Cl at a ratio (v/v) of 100:1 disinfectant to PrP^Sc^ prior to immunoblotting using R30 antiserum against PrP residues 90–104. PrP monomer (curly bracket) and PrP aggregates (square bracket) are marked. Gels shown are representative of three independent experiments. (C) ScBH was treated with saline (mock disinfectant), or active or inactive BrioHOCl solutions at a ratio (v/v) of 10:1 disinfectant to 10% ScBH for the designated time and analyzed by SDS-PAGE with Deep Purple total protein stain. Insoluble aggregates are indicated with an arrowhead. (D) The samples from panel C were analyzed by immunoblot using R30 PrP antiserum. Full length, diglycosylated PrP monomer (double arrowhead); Truncated, and/or less glycosylated PrP monomer (single arrowhead) are identified.

To test for molecular effects of BrioHOCl we used SDS-PAGE gels to examine PrP^Sc^ after exposure to BrioHOCl. First, we tested the effects of increasing incubation times with purified PrP^Sc^. A broad-spectrum protein stain indicated that as little as 1 min exposure to 10-fold volumes of BrioHOCl resulted in the reduction of detectable SDS-solubilized PrP^Sc^ monomer (curly bracket; Fig 8A) and an increase in high molecular weight SDS-resistant species, including protein species at the top that were excluded from the gel (arrowhead; Fig 8A). These effects were coincident with the rapid reduction of prion seeding activity seen with treatment of prion-infected BH prior to RT-QuIC analysis (Fig 5). Western blot analysis showed that BrioHOCl reduced the amount of SDS-solubilized PrP^Sc^ monomer (curly bracket) and increased high molecular weight species (square bracket) detectable with a PrP antiserum directed at PrP residues 90–104 (Fig 8B). These effects were most apparent with BrioHOCl solutions containing ≥160 ppm Cl.

We also treated ScBH with 10-fold volumes of BrioHOCl. Total protein staining indicated that treatment has an immediate (≤1 min) and broad effect on all proteins, resulting in the loss of multiple individual protein bands and the accumulation of high molecular weight species (Fig 8C) consistent with previous observations [53]. Western blot analysis with the PrP antiserum indicated a preferential loss of the full-length, diglycosylated PrP^Sc^ monomer band (double arrowhead; Fig 8D). It is unclear if the loss of this band reflects a truncation of full-length PrP^Sc^ or preferential modification to the diglycosylated PrP^Sc^, possibly due to the increased solvent accessibility of one glycan over the other. The accumulation of high molecular weight PrP species seen with purified PrP^Sc^ in Fig 8B was not recapitulated here, but we assume that this was due to competition with the transfer of aggregated PrP molecules to the blotting membranes by the vast excess of other aggregated proteins in the HOCl-treated crude ScBH shown in Fig 8C.

### Effects of BrioHOCl treatments on α-synuclein seeds

**Fig 9:**
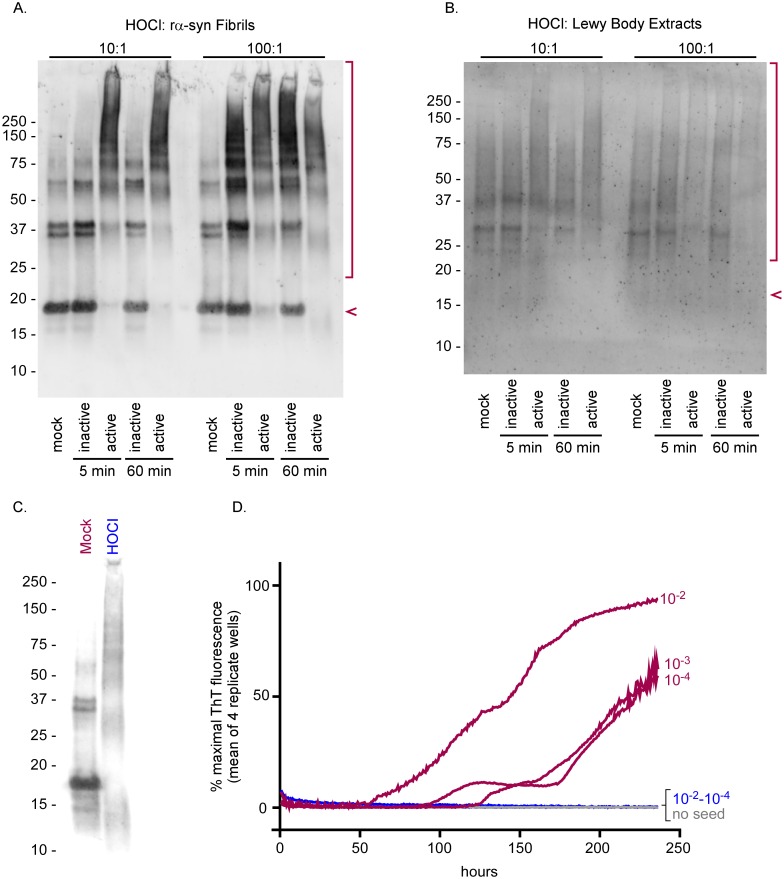
Effects of BrioHOCl on recombinant α-synuclein seeds and Lewy bodies. Recombinant α-syn (rα-syn) fibrils generated *in vitro* (A) or Lewy Bodies isolated from a patient with Lewy Body Dementia (B) were treated with an active (190 ppm Cl) or inactive (30 ppm Cl) BrioHOCl solution at a 10:1 or 100:1 disinfectant to α-syn ratio for 5 or 60 min, as indicated, and probed for α-syn by immunoblot. Samples treated 10:1 were diluted an additional 10 fold in 1x sample buffer prior to loading the gel to match the protein concentrations of the 100:1 treated samples on the immunoblot. The arrow indicates monomeric α-syn protein and the bracket denotes aggregates and degradation products (A & B). Recombinant α-syn fibrils generated in vitro were treated with either a mock solution or an active BrioHOCl solution at 100:1 disinfectant to α-syn ratio for 60 min and probed for α-syn by immunoblot (C). These mock (red) and HOCl (blue) treated samples were subjected to 10 fold serial dilutions and analyzed for recombinant α-syn seeding activity (D). 20 μl per well of 10^−2^ through 10^−4^ sample dilutions were used as reaction seeds as indicated. Negative control reactions were run with no seed (gray). Other controls indicated that direct addition of 10^−3^ and 10^−4^ dilutions of BrioHOCl to the seeded polymerization reactions without preincubation with the α-syn seed had no effect on the reaction kinetics, whereas a 10^−2^ dilution partially interfered with the reaction (S3 Fig). Each trace represents the average ThT fluorescence of 4 replicate wells. Similar results were obtained in two additional independent experiments.

Given that many other proteins besides PrP can form pathological amyloids or oligomers with apparent “prion-like” seeding activity, we investigated whether HOCl solutions might also inactivate seeds composed of other amyloid proteins. α-Synuclein is the main protein included in the amyloid fibrils that accumulate in various synucleinopathies such as Parkinson’s disease [54]. As was seen above with PrP^Sc^ and other proteins, BrioHOCl treatments of synthetic recombinant α-synuclein amyloid fibrils (rα-syn fibrils), as well as α-synuclein-containing Lewy bodies isolated from brain tissue of a Lewy body dementia patient, reduced the detection of α-synuclein monomers and enhanced the detection of higher-order SDS-insoluble aggregates (Fig 9A and 9B). However, for unknown reasons the latter effect was less apparent with the Lewy body extracts (Fig 9B). rα-Syn fibrils showed this effect after 5-min treatments with a BrioHOCl preparation previously shown to be active against prion seeding activity (Fig 9A). A preparation previously shown to be inactive in inhibiting prion seeding activity was also inactive against rα-syn fibrils (Fig 9A). Similar effects were seen with the treatment of Lewy body extracts from brain tissue, however a longer treatment and higher HOCl:rα-syn fibril ratio were needed to cause a visible effect (Fig 9B).

To investigate the effect of BrioHOCl treatment on the α-syn seeding activity rα-syn fibrils treated with BrioHOCl or 100mM Tris-HCl (Mock) (Fig 9C) were used to seed the polymerization of soluble recombinant α-synuclein. Mock treated fibrils were capable of seeding with 10^−2^–10^−4^ dilutions (Fig 9D). All seeding activity was abolished in BrioHOCl-treated fibrils.

### Effects of BrioHOCl treatments on tau peptide seeds

**Fig 10:**
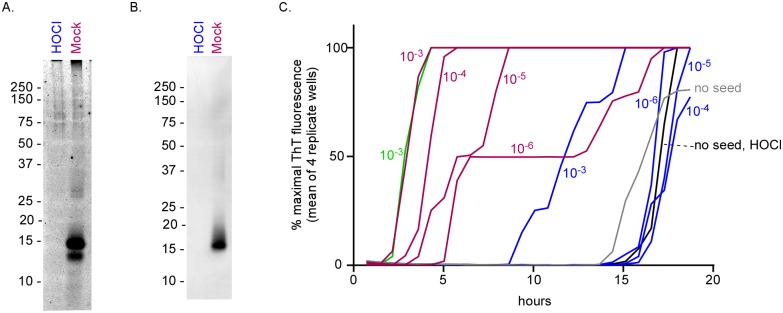
Inactivation of tau peptide amyloid seeds by BrioHOCl. Synthetic tau seeds were generated with recombinant K19 Cys-free tau fragment and treated with or without a 100-fold excess of BrioHOCl. The seed preparations were subjected to SDS-PAGE with non-specific staining for protein (Deep Purple) (A), immunoblotting probed with anti-tau antibody (B) or a seeded polymerization assay (C). In the seeded polymerization assay, the designated dilutions of the untreated (red) or BrioHOCl-treated (blue) seed samples were tested. To control for potential effects of BrioHOCl on the assay itself without allowing time for prior interactions the amyloid seed on its own, an amount of BrioHOCl comparable to that in a 10^−3^ dilution of treated seed was added directly to reactions solutions that were either left unseeded (black) or seeded with a 10^−3^ dilution of untreated seed (green). Reactions mock-seeded with 10^−5^ dilutions of NBH samples that were treated or not with 20 or 100 volumes of BrioHOCl are all shown in gray. Similar effects on K19 Cys-free seeding activity were obtained in at least five independent experiments.

We also tested effects of HOCl on synthetic amyloid seeds composed of a fragment of human tau. Amyloid seeds were prepared from a recombinant tau fragment (“K19 Cys-free”; residues 244–372 with Cys322 mutated to serine [55]). Treatment of these seeds with a 100-fold excess of BrioHOCl markedly reduced detection of intact K19 Cys-free peptide by either non-specific protein staining (Fig 10A) or immunoblotting (Fig 10B). However, unlike the above observations with other proteins, we did not detect any increases in aggregated tau peptide in the upper parts of the gel lanes. Nevertheless, the BrioHOCl treatment markedly increased the lag phases of seeded polymerization of soluble K19 Cys-free substrate (Fig 10C). The HOCl-treated 10^−3^ dilution of the K19 Cys-free tau seed gave lag phases that were at least as long as the 10^−6^ dilution of the untreated seed, while the lag phases from further dilutions of the HOCl-treated seed were indistinguishable from spontaneous (unseeded) polymerization. Comparisons of these relative lag phases indicated that BrioHOCl reduced the seeding activity by at least 1,000-fold.

## Discussion

Numerous clinical and agricultural scenarios involving potential prion contamination would benefit from the availability of less harsh and more practical methods for inactivating prions. Here, we have demonstrated that weakly acidic BrioHOCl has strong anti-prion activity. In the case of hamster ScBH, we have shown a reduction in infectivity titer of ≥~10^5.75^-fold by bioassay in mice (Table 1). This reduction is commensurate with the decrease in prion seeding activity measured by RT-QuIC. For sCJD, vCJD, C-BSE, CWD and sheep scrapie we have shown that BrioHOCl eliminates all detectable RT-QuIC seeding activity (Fig 2). Although we have not also done animal bioassays of BrioHOCl-treated brain homogenates containing these other strains, many prior studies have indicated that prion infection and RT-QuIC positivity in *ex vivo* samples are strongly correlated, and that RT-QuIC is at least as sensitive analytically as animal bioassays [32, 36, 37, 42, 43]. These results imply so far that if a disinfectant eliminates RT-QuIC seeding activity, it will likely also eliminate prion infectivity.

However, we should note that the inverse is not necessarily true; that is, elimination of prion infectivity might not always be accompanied by loss of RT-QuIC seeding activity. For example, Environ LpH^™^ is much more effective at decreasing bioassayed scrapie infectivity ([16];Tables 1 and 2) than RT-QuIC seeding activity. This is not surprising given that synthetic recombinant PrP amyloids can have RT-QuIC seeding activity but no apparent infectivity in animals. Thus, infectious PrP^Sc^ comprises only a subset of PrP particles with RT-QuIC seeding activity, so treatments may neutralize the infectivity of PrP^Sc^ without proportionally affecting all possible types of PrP seeding activity. Accordingly, in future potential applications of RT-QuIC assays in screening for other anti-prion treatments, it would be advisable to use infectious tissue-derived prions/PrP^Sc^ as test specimens rather than non-infectious synthetic amyloid seeds. Although hits from such screens against PrP^Sc^ are likely to be effective against infectivity, it is possible that treatments that can neutralize infectivity without eliminating RT-QuIC seeding activity, like Environ LpH^™^, would be missed.

From a mechanistic perspective, we have shown a positive dose-response relationship between the free Cl concentrations of BrioHOCl preparations and prion seed inactivation (Fig 6, Table 3), protein aggregation, and loss of detectable SDS-soluble protein monomers (Fig 8). The presence of Raman spectroscopy signals for HOCl but not hypochlorite ion, aqueous chlorine, or any other species that may arise during electrolysis of NaCl solutions [56] is consistent with the observed effects being attributable to HOCl. However, it remains possible that other as yet unidentified constituents or characteristics resulting from the manufacturing process contribute to the unusual stability and inactivating activity of BrioHOCl.

Despite the high probability that our observations result primarily from the activity of HOCl, we cannot yet pinpoint a particular anti-prion mechanism of BrioHOCl. HOCl can covalently modify a number of different amino acid side chain moieties on proteins, including thiols, amines, aromatic amino acids, and backbone peptide bonds. HOCl reacts most rapidly with sulfur-containing amino acids. Oxidation of methionine leads to the formation of sulfoxide, while disulfide bonds and oxy-acids are the products of cysteine oxidation [57]. Chlorination of lysines and tyrosines leads to formation of chloramines, and 3-Cl-tyrosine and 3,5-Cl-tyrosine residues, respectively. Tyrosines can also undergo dimerization via the formation of phenoxyl radicals, leading to protein crosslinking [58, 59]. Although less reactive than many amino acid side chains, backbone amide bonds can be chlorinated by excess HOCl leading to polypeptide fragmentation [60]. Any of these modifications, including those leading to further PrP^Sc^ aggregation by crosslinking, may modify or occlude seeding surfaces on PrP^Sc^ even without unfolding the protein, preventing PrP^Sc^ from converting PrP monomers into more PrP^Sc^ *in vivo*, or into recombinant PrP amyloid *in vitro*. A loss of specific infectivity with further aggregation of PrP^Sc^ would be consistent with previous observations that the most infectious prion particles are small and non-fibrillar [61].

We have shown activity of a HOCl formulation against prions in wet tissue homogenates and dried onto stainless steel wires. The ability to inactivate prions on stainless steel implements and instruments without damaging them would reduce risks of iatrogenic transmission in clinical settings, autopsy rooms and slaughter houses. Further work will be required to ascertain whether BrioHOCl can not only inactivate prions bound to stainless steel, but also to other types of materials such as those covering gastroscopes, broncoscopes and rhinoscopes.

Although our results indicate that BrioHOCl’s active Cl content can decline over time, its stability on storage in sealed high density polyethylene vessels (half-life estimated to be 440 days), even under less-than-ideal warehouse storage conditions, is compatible with various practical applications. Certainly, further work is required to optimize storage vessel selection and fluid handling. Nevertheless, the speed and efficacy of prion inactivation even with test samples from barreled production lots >9 months old (Figs 1, 2, 4 and 5; Tables 1 and 2), together with the persistence of high level and rapid efficacy (4–7 LRV) against some of the most resistant microbes known (*Bacillus* and *Aspergillus* spores) (S1 Table), support the practical utility of BrioHOCl. Given the prolonged persistence of prions in the environment (e.g. [62–64]), it is important to have practical means of neutralizing prion infectivity in natural settings as well as procedure rooms, operating rooms, animal handling facilities and food processing plants.

The generality of the effects of this HOCl formulation on proteins, as evidenced by the mobility shifts of many brain homogenate proteins (Fig 8), is consistent with its effects on PrP^Sc^ (Fig 8) and amyloid seeds of α-synuclein (Fig 9) and the tau fragment (Fig 10). This raises the intriguing possibility that HOCl could have even broader effects on pathological protein aggregates that are capable of seeding their own accumulation. Recent studies have indicated experimental transmissibilities of several protein misfolding processes such as those of Alzheimer disease, Parkinson disease, multiple systems atrophy [65, 66], and tauopathies (reviewed in [9, 67]). Although to our knowledge there is no clear evidence of transmissions of these diseases between humans, these studies have raised concerns that self-propagating protein amyloids, e.g. those composed of Aβ [68], α-synuclein, and tau, might pose risks of iatrogenic transmission via contaminated medical instruments or transplanted tissues. If the seeding activity associated with these various diseases can be inactivated by appropriate HOCl exposure as suggested by our study, then such potential transmission risks might be mitigated.

Finally, as noted above, HOCl is produced naturally *in vivo* by a variety of “professional” phagocytes such as neutrophils, microglia and macrophages as part of innate immune mechanisms to inactivate microbial pathogens and trigger a variety of beneficial pathophysiological responses to injury. The fact that we have now shown that HOCl also has anti-prion activity *in vitro* raises the possibility that the same might be true *in vivo*. Many proteins can form misfolded oligomers and amyloid fibrils that can seed their own growth and accumulate in tissues to cause pathological changes. Protein quality control systems such as the unfolded protein response, chaperones, ubiquitination, proteasomes and autophagy can usually prevent the accumulation of misfolded proteins within cells [69]. However, it is less clear how organisms ordinarily cope with amyloid-like aggregates that escape these systems and accumulate inside or outside cells. Perhaps such aggregates can be recognized, and exposed to HOCl or other more stable products of the reactive oxygen burst such as N-chlorotaurine [70]. Such HOCl exposure might inactivate the self-propagating activity of protein aggregates and/or aid in their clearance. Further studies will be required to evaluate whether such a mechanism is a significant component of proteostasis *in vivo*.

## Materials and Methods

### Anti-Prion reagents

Pure Bright Germicidal Ultra Bleach^®^ was used as the source for Na hypochlorite (6%). Environ LpH^™^ was obtained originally from Steris Inc. and had been stored for ≥6 years prior to use. It should be noted that this specific Environ LpH^™^ formulation differs from product sold with the same name in Europe [17]. BrioHOCl was produced from a saturated NaCl solution and filtered water in an electrochemical cell by a proprietary process of Briotech Inc., Woodinville, WA. We confirmed that HOCl is a primary active component of BrioHOCl, and that other potential electrochemical reaction products such as OCl^-^ (hypochlorite) or molecular chlorine (Cl_2_) were undetectable, by Raman spectroscopy (S1 Fig).

### Disinfectant treatments of BH suspensions

10% (w/v) brain homogenates (BH), defined as 10^−1^ tissue dilutions, were prepared as described previously [32] from brain tissue obtained from a scrapie-infected hamster, a BSE-infected cow (a gift from Dr. Kentaro Masujin, National Institute for Animal Health, Tsukuba, Japan), a scrapie-infected sheep (a gift from Dr. David Schneider, Animal Disease Research Unit, USDA-ARS, Pullman, WA), a human vCJD decedent (Drs. Kaetan Ladhani and Jillian Cooper at the CJD Resource Centre, NIBSC, Herts, UK), and a CWD-infected mule deer (Drs. Michael Miller, Colorado Department of Wildlife; Elizabeth Williams and Jean Jewell, University of Wyoming; and Terry Kreeger, Wyoming Game and Fish Department). In each case, 10% BH (w/v) was incubated at room temperature for the designated time in saline (0.9% NaCl), BrioHOCl, 1M NaOH, 40% household bleach (2.4% Na hypochlorite) or 2% Environ LpH^™^ at a ratio of 100:1, or 20:1 (v/v, disinfectant:BH) as specified. Following incubation, serial 10-fold dilutions were prepared in Sample Diluent (PBS, 0.1% SDS and Gibco N2 media supplement) and seeding activity was quantified by end-point dilution RT-QuIC analysis followed by Spearman-Kärber analysis to estimate the seeding dose giving positive reactions in 50% of the technical replicates [32]. In other experiments, a milder deactivation condition was used in which 10% (w/v) BH was incubated for only 5 min in saline or the designated disinfectants and then immediately diluted into Sample Diluent to greatly reduce continued effects of the disinfectants.

### Disinfection treatments of scrapie-coated wires

Batches of sterile stainless steel suture wire (Havel, size 000), cut into 3–4mm lengths, were soaked in BH from normal (uninfected) or scrapie-infected animals at tissue dilutions of 10^−3^–10^−10^ for 1 h at room temperature, washed 3 times with a brief vortex in 1ml PBS, and left to dry in a sterile petri dish. Additional batches of wires coated with 10^−3^ scrapie-infected BH (ScBH) were further treated by submerging in disinfectants (BrioHOCl, 1M NaOH, 40% household bleach (2.4% hypochlorite), 2% Environ LpH^™^ or saline (mock disinfectant)).

### RT-QuIC analyses

All RT-QuIC seeding assays were conducted using conditions similar to those described previously (eg. [32]) with variations described below. Hamster (90–231) recombinant prion protein (rPrP^C^) (Accession number K02234) or chimeric hamster-sheep rPrP^C^ (Ha-S; Syrian hamster residues 23 to 137 followed by sheep residues 141 to 234 of the R_154_Q_171_ polymorph [accession nos. K02234 and AY907689] [71]) were used as substrates in RT-QuIC experiments as indicated. Purification of hamster (90–231) and Ha-S rPrP^C^ was conducted as previously described [72].

To measure prion seeding activity in brain tissue dilutions, 2 μl of each dilution was added to 98 μl RT-QuIC reaction solution to give final concentrations of 0.1 mg/ml rPrP^C^, 10 mM phosphate buffer (pH 7.4), 10 μM thioflavin T (ThT), 300 mM NaCl, 1 mM EDTA and 0.002% SDS. This final concentration of SDS in the reaction volume resulted from dilution of seed sample containing 0.1% SDS. Four technical replicate reaction wells at each dilution were set up in a 96-well plate. For analysis of wires, single wires were transferred into wells containing 100 μl of the RT-QuIC reaction solution with the 0.002% SDS final concentration added directly. The plates were then shaken in a temperature-controlled fluorescence plate reader (BMG FLUOstar) at 42°C unless indicated otherwise with cycles of 1 min double orbital shaking at 700 rpm and 1 min of rest [32]. ThT fluorescence was measured at 45-min intervals.

To measure α-syn seeding activity recombinant α-syn purchased from rPeptide (Catalog # S 1001 1) was used as a substrate. α-Syn fibrils were generated in 20 mM Tris-HCl, 100 mM NaCl, pH 7.4 through constant shaking at 1000 rpm while incubating at 37°C for 5 d in a Eppendorf Thermomixer R. 20 μL of these fibrils, or fibrils treated with HOCl as described, were used to seed a reaction mix containing final concentrations of 104 mM Tris, 20 mM NaCl, 10 μM ThT, and 30 μM α-syn, at pH 7.5. Seeded reactions were incubated at 37°C with the shake-rest cycles and reading parameters the same as for RT-QuIC.

For tau-based RT-QuIC reactions, the cysteine-free K19 tau fragment was expressed and purified as previously described [55, 73] with modifications. Synthetic tau seed was generated and seeding assays with fluorescence detection were performed in HEPES-buffered saline solutions containing low molecular weight heparin by following the protocol described [55] with modifications such as periodic shaking in a 96-well plate rather than sonication in tubes.

All RT-QuIC experiments were set up such that the plate readers would give a ThT fluorescence negative control baseline of around 50,000 rfu (relative fluorescence units). These readers have a fluorescence saturation signal of 260,000 rfu. Following collection the experimental data was normalized such that the baseline signal of the lowest negative control was set at 0% and the saturation signal of 260,000 rfu was set at 100%. The individual traces graphed are the averages of the 4 wells for each dilution tested. For Spearman-Kärber analyses of end-point dilution RT-QuIC experiments [32, 74], individual reaction wells were judged to be positive at 50 h when the signal exceeded 50% of the saturation signal. The seeding dose (± S.E.) giving ThT positivity in 50% of technical replicate wells (SD_50_) was calculated as described using the S.E. “smoothing” procedure to account for small group size [68].

### Mice

Homozygous, tg7 mice on a C57BL/10 background were bred at RML and used for all bioassay experiments. Creation of the original tg7 mice has been described previously [31]. The tg7 mice used in these bioassays over-express hamster PrP (approximately 5-fold) under the control of the endogenous mouse PrP promoter and do not express any mouse PrP.

### Mouse bioassay of tissue suspensions

Following the 100:1 *Disinfectant treatments of BH suspensions* described above a 10^−3^ dilution of ScBH was further diluted in serial 10-fold increments into PBS for inoculation into mice. The following dilutions of treated ScBH were tested: Saline treatment group, 10^−4^ through 10^−10^; BrioHOCl, 10^−3^ through 10^−8^; NaOH, 10^−5^ through 10^−8^; bleach, 10^−5^ through 10^−8^; Environ LpH^™^, 10^−5^ through 10^−9^. The dilutions selected for bioassay were based on expected levels of infectivity or, in some situations, the disinfectants were further diluted prior to inoculation to prevent acute toxicity (i.e., for NaOH, bleach and Environ LpH^™^). Each dilution was inoculated intracerebrally into groups of 4 tg7 mice. For the inoculation, mice were anesthetized with isoflurane and inoculated in the left brain hemisphere with 30 μl of dilutions of disinfectant- or saline-treated ScBH. Following inoculation mice were monitored for onset of scrapie. Mice were euthanized when they displayed advanced stages of scrapie including poor grooming, kyphosis, ataxia, wasting, delayed response to stimuli, and somnolence. Following euthanasia brains were removed and flash frozen for biochemical analysis. Infectivity titers were calculated for each experimental group using the Spearman-Kärber formula [32].

### Mouse bioassay of steel-bound scrapie infectivity

For wire implantation, experimental groups were 3–8 mice (Table 2). Tg7 mice were anesthetized with isoflurane gas and the dorsal surface of the mouse skull was shaved, ophthalmic ointment was applied to protect each eye, and the dorsal surface of the skull was scrubbed with chlorhexidine surgical scrub. Each mouse was then positioned in a stereotactic device and isoflurane anesthesia was provided via nose cone. Using aseptic technique a midline incision was made on the skin of the skull to expose the bregma landmark. The drill was positioned at a location 1 mm anterior to bregma and 1.7 mm to the left, lateral side of midline (above the striatum). A small hole was drilled at this location and a 3–4 mm pre-treated stainless steel wire was inserted. Bone wax was used to seal the defect in the skull once the wire was is in place. The incision was closed with 5–0 PDS suture in a cruciate pattern. Mice were placed in heated cages following surgery until fully recovered. Each mouse received 0.2 mg/kg buprenorphine (Buprenex) subcutaneously immediately post-surgery. Following implantation tg7 mice were monitored for onset of scrapie. Mice that developed disease were euthanized. At the time of euthanasia, all the wires were confirmed to be in place and showed no signs of deterioration.

### Active chlorine and other BrioHOCl characteristics

Hach reagent kits for Total (active, free) Chlorine (Hach Company, Loveland, CO) were used for determination of the active Cl content of the BrioHOCl formulation, after validation by comparison of manual iodometric and digital titration results on 33 samples (6 replicates each). Thereafter the digital Hach device was used (4 replicates per sample) to measure active Cl in all samples used for inactivation experiments with PrP^Sc^, and for antimicrobial efficacy testing. Titratable free Cl concentrations were also measured in archived samples at Briotech (oldest 34 months), and, to establish the Cl trends, in a serially sampled lot of BrioHOCl. The latter were stored in sealed ~100 mL aliquots in HDPE bottles at 21°C, and prepared specifically for this purpose. All other HOCl samples used throughout this study were derived from routine production electrolysis runs at the manufacturing plant. Product from each lot was stored in different vessel types (100 mL up to 4 L bottles, and 220 L barrels, all HDPE) in uncontrolled temperature warehouse environments (3.5°C to 35°C). Small vessels were sealed with aluminum caps, and drums lids were tightly sealed to avoid exposure to air (known to be deleterious), but no optimization of storage conditions was attempted for materials used herein. The pH, oxidation-reduction potential (ORP, in mV) and conductivity were recorded for all samples using a Hach Multi Parameter meter (Model HQ40d). ORP targeted at production was +1140 mV, at pH 3.9. Starting active Cl concentrations were varied in production lots during electrolysis, depending on intended applications. Generally these values ranged between 175 and 350 ppm active Cl, with background NaCl concentrations of either 0.9 or 1.8%. Solutions with both NaCl backgrounds were tested in RT-QulC assays.

### UV/Vis spectrophotometry

Test solutions were loaded into 1 mL quartz cuvettes, and spectra obtained using a BioMate 3S UV-Visible Spectrophotometer. The instrument was blanked using Nanopure water, and test solutions consisted of undiluted BrioHOCl at selected time points in the sequential sampling of product stored at room temperature. Absorbance was measured from 190–400 nm.

### Raman spectroscopy

Spectra were obtained using a Renishaw InVia Raman microscope. Spectra were observed using an excitation wavelength of 785 nm with undiluted BrioHOCl in a 1 mL quartz cuvette. The acquisition time for each scan was 20 s, and 100 acquisitions were accumulated. A deionized water blank was scanned in the same manner, and subtracted from the test sample data using Igor software (WaveMetrics).

### Protein gel analysis

PrP^Sc^, purified as previously described [75], hamster ScBH at a dilution of 10^−1^, α-synuclein, or Lewy bodies isolated from the brains of a Lewy body dementia patient, as described [76], were pretreated with saline or BrioHOCl solutions as indicated in the figure legends. Following treatment, samples were diluted with equal volumes of 2X sample buffer (125 mM Tris-HCl pH 6.8, 5% glycerol, 6 mM EDTA, 10% SDS, 0.04% bromophenol blue, 48% urea, 8% 2-mercaptoethanol) and boiled for 5 min. Equal volumes of samples were run on 10% Bis-Tris NuPAGE gels (Invitrogen) and used for subsequent Deep Purple protein stain per manufacturer’s instructions (GE Healthcare) or the protein transferred to an Immobilon P membrane (Millipore) using the iBlot Gel Transfer System (Life Technologies). PrP was detected using rabbit PrP antisera R30 (1:10,000; residues 90–104) [77, 78] and alkaline-phosphatase conjugated secondary antibody (1:5000; Jackson ImmunoResearch). α-Synuclein was detected using mouse Anti-α-Synuclein Clone 42 antibody (1:1000; BD Transduction Laboratories) and alkaline-phosphatase conjugated secondary antibody (1:2000; Jackson ImmunoResearch). Tau was detected using a tau antibody (anti-tau ab64193, Abcam) as the primary antibody.

### Ethics statement

All mice were housed at the Rocky Mountain Laboratory (RML) in an AAALAC-accredited facility in compliance with guidelines provided by the Guide for the Care and Use of Laboratory Animals (Institute for Laboratory Animal Research Council). Experimentation followed RML Animal Care and Use Committee approved protocol #2015–070 in compliance with guidelines provided by the Guide for the Care and Use of Laboratory Animals (Institute for Laboratory Animal Research Council).

## Supporting Information

S1 Fig Raman spectroscopy of BrioHOCl. (DOCX)

S1 Table Antimicrobial efficacy of BrioHOCl in ASTM E2315 Time vs. Kill suspension test protocol using lot samples of different ages. (DOCX)

S2 Fig RT-QuIC seeding activity tolerance for BrioHOCl. RT-QuIC analysis was performed with Hamster (90–231) recombinant prion protein substrate at 42°C using 2μl per well of normal brain homogenate (gray) or hamster scrapie brain homogenate at a tissue dilution of 5x10^-8^ as reaction seed in the presence of 0 (red) or 0.001, 0.01, 0.1, 1 & 10% BrioHOCl (blue) is indicated. In each case BrioHOCl concentrations were added directly to the reaction volume in the wells. Each trace represents the average ThT fluorescence of four technical replicate wells normalized between baseline and maximal signal and graphed here as a function of time.
(DOCX)

S3 Fig Tolerance of α-synuclein RT-QuIC assay for BrioHOCl. Direct addition of BrioHOCl to α-synuclein RT-QuIC reactions seeded with a 10^-2^ dilution of an artificial α-syn seed. While direct addition of the equivalent to a 10^-2^ dilution (1.8% HOCl, blue line) partially interfered with the reaction (compared to the no HOCl control, orange line), 10^-3^ (0.18%), 10^-4^ (0.018%), and 10^-5^ (0.0018%) dilution equivalents of HOCl had no effect on the reaction kinetics when directly added to the reaction without preincubation with the α-syn seed.
(DOCX)
