# Supplementary material for: Inactivation of Prions and Amyloid Seeds with Hypochlorous Acid
Source: PLoS Pathog. 2016 Sep 29;12(9):e1005914. doi: 10.1371/journal.ppat.1005914 (PMC5042475; doi:10.1371/journal.ppat.1005914)
Supplement: S1 Fig — (DOCX) [file ppat.1005914.s001.docx]

**Raman spectroscopic analysis of Briotech HOCl preparations**

In freshly prepared BrioHOCl, and in a sample produced 14 months before analysis of the same lot (#1504601) used to demonstrate *in vivo* and *in vitro* anti prion effects over a 9 month period at RML, Raman spectroscopy revealed a peak at 728 cm^-1^ (S1 Fig) corresponding to HOCl rather than ClO^-^ (715 cm^-1^) [27, 56]. Cl_2_ would have given a peak at ~538 cm^-1^ [27].

**S1 Fig. Raman spectroscopy of BrioHOCl.**
