# Supplementary material for: Inactivation of Prions and Amyloid Seeds with Hypochlorous Acid
Source: PLoS Pathog. 2016 Sep 29;12(9):e1005914. doi: 10.1371/journal.ppat.1005914 (PMC5042475; doi:10.1371/journal.ppat.1005914)

**Antimicrobial properties of BrioHOCl**

As other preparations of hypochlorous acid are well known to have microbicidal activity, we tested if the same is true of BrioHOCl formulations used for anti-prion testing. Test samples of BrioHOCl varying in age from 3 to 34 months showed high efficacies in inactivating a range of target microbes. Exposures as brief as 15-20 sec were generally sufficient to produce LRVs in the 4-7 range, for all microbes tested, with the potency declining noticeably in the oldest materials tested. Aging of the samples was associated with lower titratable active Cl concentrations. *Aspergillus* spores proved the least susceptible to inactivation by BrioHOCl, although even in that case exposures of 60 sec resulted in an LRV of >6 with the freshest, 3-month-old sample of BrioHOCl. Over time in storage the pH of the formulation trended upwards from the starting production-targeted level of 3.9 to ~5 by the second year, regardless of the type of storage vessel (PTFE 4 oz bottles, gallon jugs, or 55 gallon barrels).

**S1 Table**


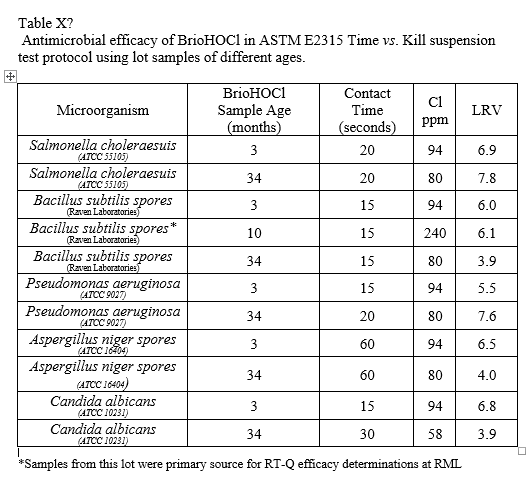

Supplement: S1 Table — (DOCX) [file ppat.1005914.s002.docx]
