# Supplementary material for: Inactivation of Prions and Amyloid Seeds with Hypochlorous Acid
Source: PLoS Pathog. 2016 Sep 29;12(9):e1005914. doi: 10.1371/journal.ppat.1005914 (PMC5042475; doi:10.1371/journal.ppat.1005914)
Supplement: S2 Fig — RT-QuIC analysis was performed with Hamster (90–231) recombinant prion protein substrate at 42°C using 2μl per well of normal brain homogenate (gray) or hamster scrapie brain homogenate at a tissue dilution of 5x10^-8^ as reaction seed in the presence of 0 (red) or 0.001, 0.01, 0.1, 1 & 10% BrioHOCl (blue) is indicated. In each case BrioHOCl concentrations were added directly to the reaction volume in the wells. Each trace represents the average ThT fluorescence of four technical replicate wells normalized between baseline and maximal signal and graphed here as a function of time. (DOCX) [file ppat.1005914.s003.docx]

**S2 Fig. RT-QuIC seeding activity tolerance for BrioHOCl**


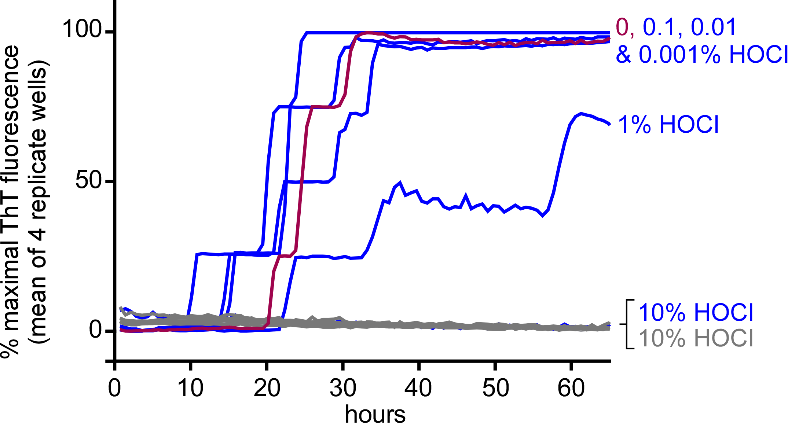


RT-QuIC analysis was performed with Hamster (90-231) recombinant prion protein substrate at 42°C using 2µl per well of normal brain homogenate (gray) or hamster scrapie brain homogenate at a tissue dilution of 5x10^-8^ as reaction seed in the presence of 0 (red) or 0.001, 0.01, 0.1, 1 & 10% BrioHOCl (blue) is indicated. In each case BrioHOCl concentrations were added directly to the reaction volume in the wells. Each trace represents the average ThT fluorescence of four technical replicate wells normalized between baseline and maximal signal and graphed here as a function of time.
