# Supplementary material for: Inactivation of Prions and Amyloid Seeds with Hypochlorous Acid
Source: PLoS Pathog. 2016 Sep 29;12(9):e1005914. doi: 10.1371/journal.ppat.1005914 (PMC5042475; doi:10.1371/journal.ppat.1005914)
Supplement: S3 Fig — Direct addition of BrioHOCl to α-synuclein RT-QuIC reactions seeded with a 10-2 dilution of an artificial α-syn seed. While direct addition of the equivalent to a 10-2 dilution (1.8% HOCl, blue line) partially interfered with the reaction (compared to the no HOCl control, orange line), 10-3 (0.18%), 10-4 (0.018%), and 10-5 (0.0018%) dilution equivalents of HOCl had no effect on the reaction kinetics when directly added to the reaction without preincubation with the α-syn seed. (DOCX) [file ppat.1005914.s004.docx]

**S3 Fig. Tolerance of α-synuclein RT-QuIC assay for BrioHOCl**

Direct addition of BrioHOCl to α-synuclein RT-QuIC reactions seeded with a 10^-2^ dilution of an artificial α-syn seed. While direct addition of the equivalent to a 10^-2^ dilution (1.8% HOCl, blue line) partially interfered with the reaction (compared to the no HOCl control, orange line), 10^-3^ (0.18%), 10^-4^ (0.018%), and 10^-5^ (0.0018%) dilution equivalents of HOCl had no effect on the reaction kinetics when directly added to the reaction without preincubation with the α-syn seed.
